# News and Events

**Published:** 2009

**Authors:** T. Ganesh

**Affiliations:** Department of Radiation Oncology, King Fahad Specialist Hospital, Dammam, Kingdom of Saudi Arabia, Saudi Arabia. E-mail: sptgnadar@yahoo.com

## United States Population has more Medical Radiation Exposure

The Collective Effective Dose in the United States (US) population more than doubled from 835,000 person-Sv to 1,870,000 person-Sv in the period between early 1980s and 2006. US citizens were exposed to more than seven times as much ionizing radiation from medical procedures as was the case in the early 1980s which resulted in the two fold increase in Collective Effective Dose of the population. The increase was primarily a result of the growth in the use of medical imaging procedures, particularly due to the higher utilization of computed tomography (CT) and nuclear medicine. These two imaging modalities alone contributed to 36% of the total radiation exposure. In 2006, medical radiation exposure contributed to 48% of the total exposure, up from 15% in early 1980s. However, the contribution of occupational exposure to collective effective dose declined from 0.25% to 0.07% during the same period. These findings were reported in the National Council on Radiation Protection and Measurements (NCRP) Report No. 160: Ionizing Radiation Exposure of the Population of the United States.

Professionals and scientists from American Association of Physicists in Medicine (AAPM) and American College of Radiology (ACR) issued clarification statements in view of these findings to alleviate the concerns they might have on the general public. They stated that the Report should not be misinterpreted as an increase in risk to the US population without also carefully considering the tremendous and undeniable benefits of medical imaging.

From: http://www.ncrponline.org/Press_Rel/Rept_160_Press_Release.pdf

http://www.ncrponline.org/PDFs/Poster.pdf

http://www.ncrponline.org/PDFs/Poster_table.pdf

http://www.aapm.org/announcements/NCRP160PressRelease.asp

http://www.acr.org/MainMenuCategories/media_room/FeaturedCategories/PressReleases/ACRResponsetoNCRPReport.aspx

## Radiation Monitors to be installed in all Indian entry ports

The Atomic Energy Regulatory Board has initiated steps to install radiation monitors in all entry ports to detect the presence of radioactive material in imported consignments.

From: The Hindu dated March 4, 2009

## High levels of uranium found in Faridkot children

Dr. Carin Smit, a clinical metal toxicologist from Johannesburg, found that the hair samples of children having deformations, from a pocket of Faridkot in Punjab, contained high levels of uranium. Of the 149 children studied, 53 showed more traces of uranium. Following the revelation in media, Punjab government ordered a probe into the abnormalities among children. A five-member committee will look into the causes of uranium strains in inmates of Faridkot's Baba Farid Centre for Special Children.

From: Times of India dated April 2 and 3, 2009

## Solar Energy Round the Clock

Mirrors, synthetic oil, and fertilizer salts combine to yield one of the cleanest sources of energy – solar energy, round the clock. Andasol 1, the solar power plant in southern Spain, uses precisely curved mirrors that follow the arc of the sun, concentrating its rays onto pipes filled with a synthetic oil to heat it up to 400°C. The oil is then used to either boil water to power steam turbines, or to pump excess heat into vats of salts (a mix of potassium and sodium nitrate), turning them a molten, lava-like consistency. Molten salts can be used to store the heat from solar radiation many hours after the sun set and then release it at will to drive turbines. Andasol 1 is now providing 50 megawatts of power, enough electricity to supply 50,000 to 60,000 homes year-round. When the entire Andasol complex comprising 3 plants is completed in 2011, it will generate enough electricity to power 150,000 households.

From: The Guardian dated April 30, 2009

## The first and only known survivor of both the Hiroshima and Nagasaki atomic bombings

More than 60 years after the atomic bombings of Japanese cities Hiroshima and Nagasaki, 93-year old Tsutomu Yamaguchi, native of Nagasaki, has been officially recognized by Japanese authorities as the first and only known survivor of both attacks. He had twice been very close to the nuclear ground zero, within 3-km radius and on both occasions he escaped alive, albeit with burn injuries. He remains in relatively good health even though many of the atomic bomb survivors prematurely died from cancer and liver disease, most likely caused by their exposure to radiation.

From: http://www.guardian.co.uk/world/2009/mar/25/hiroshima-nagasaki-survivor-japan

## Health Protection Agency Report on Protection of Pregnant Patients during Diagnostic Medical Exposures to Ionizing Radiation

In collaboration with the Royal College of Radiologists and the College of Radiographers, London, Health Protection Agency (HPA) published a report on Protection of Pregnant Patients during Diagnostic Medical Exposures to Ionizing Radiation. This document provides information on the health effects that are likely to occur in the embryo or fetus following exposure to ionizing radiation during pregnancy and practical guidance on how and when to prevent or reduce unnecessary fetal exposures.

The salient features of the report are: (i) the radiation dose to the embryo or fetus from any diagnostic procedure does not pose any risk of serious health effects; (ii) clinically justified examinations giving fetal doses up to a milligray can be carried out since the associated risks of childhood cancer are very low compared to natural occurrences; termination of pregnancy is not justified at all in such cases; (iii) for procedures resulting in fetal doses in excess of few milligray, the risk is still relatively low in absolute terms; while such procedures should be avoided on pregnant women, it should not happen at the cost of their health; in those cases involving doses in excess of milligray, termination of the pregnancy shall not be justified solely on the basis of radiation risk to the unborn child; (iv) procedures resulting in fetal doses in excess of 10 mGy should be avoided; (v) likelihood of radiation induced hereditary disease in the descendants of the unborn child exposed to radiation from diagnostic procedures during pregnancy is negligible.

The report can be downloaded using the link:

http://www.hpa.org.uk/webc/HPAwebFile/HPAweb_C/1238230848746

From: http://www.hpa.org.uk/webw/HPAweb&HPAwebStandard/HPAweb_C/1238230848780?p=1199451989432&

www.auntminnie.com/index.asp?Sec=sup&Sub=wom&Pag=dis&ItemId=85376

## International Atomic Energy Agency website on radiation protection of patients

A multi-media focus page on radiation protection of patients has been launched on International Atomic Energy Agency (IAEA) website. The multi-media page contains audio and video interviews with experts, links to articles on the subject of patient protection and information for patients and public.

From: http://www.iaea.org/NewsCenter/Focus/RadiationProtection/index.html

### International Commission on Radiological Protection publishes draft report on preventing accidental exposures in radiation therapy

International Commission on Radiological Protection (ICRP) has published a draft report on ‘Preventing Accidental Exposures From New External Beam Radiation Therapy Technologies’ focusing on minimizing the risk of accidental exposure to radiation therapy patients. The report is available for download using the link:

From: http://www.icrp.org/docs/Accidental_exposure_new_RT_techniques.pdf

## Nuclear Regulatory Commission advises to enhance safety of cesium chloride sources

While considering the February 2008 report of the National Academies, “Radiation Source Use and Replacement,” which recommended action to eliminate or replace cesium chloride sources, Nuclear Regulatory Commission (NRC) took the position that near-term replacement of cesium chloride sources in existing blood, research, and calibration irradiators was not practicable and would be harmful to the delivery of medical care, research and emergency response capabilities.

The Commission has advised to continue enhancing the security of cesium chloride radiation sources, while encouraging research and further technological developments for alternative chemical forms of cesium-137.

From: http://www.nrc.gov/reading-rm/doc-collections/news/2009/09-074.html

## Danger: “Sandbag” in the MRI Room

A patient underwent magnetic resonance imaging (MRI) with a sand bag attached to her groin to help facilitate hemostasis. The bag was pulled into the MRI coil, damaging the system. Fortunately, the patient was not injured. The cause of the incident was traced to the sand bag which contained ferromagnetic iron shavings and pellets. More information can be found at:

From: http://www.fda.gov/cdrh/medicaldevicesafety/tipsarticles/sandbag.html

## Early Diagnosis of Parkinson's Disease using X-rays

Researchers have used powerful, tightly focused beams of X-rays to penetrate human tissue samples and map the metal ions, particularly iron levels, in and around individual cells with high precision. The focused beam can give information about the form in which the iron is stored. The results showed that the distribution of metal ions in the brain tissue of Parkinson's Disease sufferers was altered by the disease process. The ultimate goal of this research is to find a method for early diagnosis so that medical treatment can begin as soon as chemical changes are detected and before the irreversible cell death takes place.

From: http://www.sciencedaily.com/releases/2009/02/090213172050.htm

## Recent Publications of Interest from IAEA

### Strategies for Clinical Implementation and Quality Management of Positron Emission Tomography Tracers

This publication presents strategies for the clinical implementation and quality management of positron emission tomography (PET) tracers. The ultra-short half-lives of PET tracers, together with the busy and demanding nature of clinical settings, add to the complexities, create a need for more robust quality management programmes. This publication focuses on the clinical setting and aims to raise awareness of the issues involved and suggest means to reduce risk.

http://www-pub.iaea.org/MTCD/publications/PDF/Pub1344_web.pdf

### Cyclotron Produced Radionuclides: Physical Characteristics and Production Methods

Technical Reports Series No. 468

The publication contains chapters on accelerator technology, theoretical considerations of nuclear reactions, the technology behind targetry, techniques on preparation of targets, irradiation of targets under high beam currents, target processing and target recovery.

http://www-pub.iaea.org/MTCD/publications/PDF/trs468_web.pdf

### Radiation Protection in Newer Medical Imaging Techniques: Computed Tomography Colonography

Safety Reports Series No. 61

The publication addresses issues associated with high patient doses received from multi-detector computed tomography (MDCT) in colonography and provides data on patient dose and risk levels, as well as information for practitioners on optimizing techniques.

http://www-pub.iaea.org/MTCD/publications/PDF/pub1367_web.pdf

### Radiation Protection in Newer Medical Imaging Techniques: Cardiac Computed Tomography

Safety Reports Series No. 60

The publication addresses issues associated with high patient doses received from MDCT in cardiac imaging and provides data on patient dose and risk levels, as well as information for practitioners on optimizing techniques.

http://www-pub.iaea.org/MTCD/publications/PDF/pub1366_web.pdf

### Nuclear Medicine in Thyroid Cancer Management: A Practical Approach

IAEA TECDOC Series No. 1608

This publication is a modern-day guide for the application of nuclear medicine in the management of thyroid cancer and is divided into three sections: evaluation, management, and long term follow-up. It details the application of such technology in all relevant aspects of clinical care.

http://www-pub.iaea.org/MTCD/publications/PDF/te_1608_web.pdf

